# Research on underwater robot ranging technology based on semantic segmentation and binocular vision

**DOI:** 10.1038/s41598-024-63017-8

**Published:** 2024-05-29

**Authors:** Qing Hu, Kekuan Wang, Fushen Ren, Zhongyang Wang

**Affiliations:** 1 Sanya Offshore Oil and Gas Research Institute, Northeast Petroleum University, Sanya, 572025 China; 2 CNPC Engineering Technology Research Company Limited, Tianjin, 300451 China

**Keywords:** Underwater robot, Binocular vision, Semantic segmentation, Ranging, Computer science, Mechanical engineering

## Abstract

Based on the principle of light refraction and binocular ranging, the underwater imaging model is obtained. It provides a theoretical basis for underwater camera calibration. In order to meet the requirement of underwater vehicle to identify and distance underwater target, a new underwater vehicle distance measurement system based on semantic segmentation and binocular vision is proposed. The system uses Deeplabv3 + to identify the underwater target captured by the binocular camera and generate the target map, which is then used for binocular ranging. Compared with the binocular ranging using the original drawing, the measurement accuracy of the proposed method has not changed, the measurement speed is increased by 30%, and the error rate is controlled within 5%, which meets the needs of underwater robot operations.

## Introduction

The ocean accounts for more than 70% of the earth's total area and is rich in biological and mineral resources. All countries are racing to study the technology related to underwater robots to speed up the development and utilization of Marine resources. Whether it is the study of Marine life and submarine environment, or the detection of submarine pipeline and the salvage operation, it is inseparable from the target location and distance measurement. Compared with sonar, laser and radar, underwater optical equipment has the advantages of low cost, easy deployment and high resolution. Binocular vision technology can accurately measure underwater objects at close range, and help underwater robots perceive and locate the surrounding environment more accurately, thus improving their efficiency and safety in underwater tasks.At present, some progress has been made in the research of underwater binocular vision ranging technology. On the one hand, researchers have improved the accuracy and robustness of ranging by optimizing algorithms such as camera calibration, image preprocessing, and stereo matching. For example, using Zhang Zhengyou calibration method or other optimization algorithms to calibrate binocular cameras accurately can eliminate the distortion and error and improve the ranging accuracy. At the same time, in view of the special properties of underwater images, researchers have also proposed a variety of image enhancement and denoising methods to improve image quality and provide a better basis for subsequent stereo matching and ranging. On the other hand, with the continuous development of artificial intelligence technologies such as deep learning, underwater binocular visual ranging technology has also begun to be combined with these advanced technologies^1,2^.By training deep neural networks for feature extraction and matching, the accuracy and efficiency of ranging can be further improved. In addition, deep learning can also be used to deal with complex lighting conditions and noise disturbances in underwater environments, improving the robustness of the system.

The deep learning method has stronger flexibility and adaptability in dealing with the binocular vision ranging problem under water, and can automatically learn the strategy of extracting features and matching from images without too much manual intervention. However, deep learning methods often require large amounts of labeled data for training, and the model is complex and computationally expensive. Especially in underwater environments, obtaining high-quality, diverse annotated data can be a challenge. If the training data is insufficient or unevenly distributed, the model may have poor generalization ability and be difficult to adapt to different underwater scenes.

In contrast, stereo matching algorithm may have poor matching effect due to the change of lighting conditions and the loss of texture, and requires a lot of calculation to find the optimal matching result, which may lead to a slow running speed of the algorithm and difficult to meet the real-time requirements. However, it performs well in some scenarios and is more versatile and practical. The stereo matching algorithm calculates the depth information according to the parallax principle by directly comparing the corresponding points of the left and right views. This approach is intuitive and easy to understand, making it easy for engineers and researchers to debug and optimize. In the case of proper optimization, the stereo matching method can achieve high computational efficiency, especially when dealing with fixed mode scenes. In addition, stereo matching methods typically do not require large amounts of training data, so they may be more practical in some applications.

Deep learning method and stereo matching algorithm have unique advantages in underwater binocular vision ranging, but there are also some disadvantages. In practical application, it is necessary to choose the appropriate method according to the specific application scenario and demand, and take corresponding measures to overcome its disadvantages, so as to improve the accuracy and reliability of ranging.

Zhang^3^ proposed a target recognition and ranging system, built a convolutional neural network model, used image processing technology to identify the target and the triangular similarity principle to calculate the target distance, and finally achieved the purpose of recognition and detection. Yang^4^ used SGBM(Semi-Global Block Matching) stereo matching algorithm to enhance image contrast, reduce the influence of image color spots, ensure the robustness of the algorithm, and improve the matching search speed. Xu^5^ proposed a binocular vision guidance method, which used the image adaptive binarization algorithm and the pseudo-light source elimination algorithm to obtain the pixel coordinates of the light source identification center of the left and right images, extracted and matched the light source features, completed the accurate matching of the light source in the left and right images, and finally conducted three-dimensional ranging of the light source array.Liu^6^ proposed a novel underwater binocular depth sensing imaging element optical device. The advantage of binocular lens is that it does not require distortion correction or camera calibration. With deep-learning support, this stereo vision system can realize the fast underwater object’s depth and image computation for real-time processing capability. Meanwhile,He^7^ also proposed a stereo vision meta-lens imaging system for assisted driving vision, a comprehensive perception including imaging, object detection, instance segmentation, and depth information. The assisted driving vision provides multimodal perception by integrating the raw image, instance labels, bounding boxes, segmentation masks in depth pseudo color, and depth information for each detected object.

In this paper, a binocular vision ranging method based on semantic segmentation image is proposed by taking full advantage of deep learning and stereo matching algorithm. The method uses Deeplabv3+ semantic segmentation model to segment the object, and removes the background and retains only the object region. In the process of stereo matching, by limiting the search range of parallax to the object segmentation region, the calculation burden of irrelevant region is reduced, and the speed of binocular vision ranging is effectively improved. The experimental results show that compared with the binocular measurement results using the original image, the measurement speed is increased by nearly 30% while the measurement accuracy is unchanged.

### Binocular ranging principle

**Figure 1:**
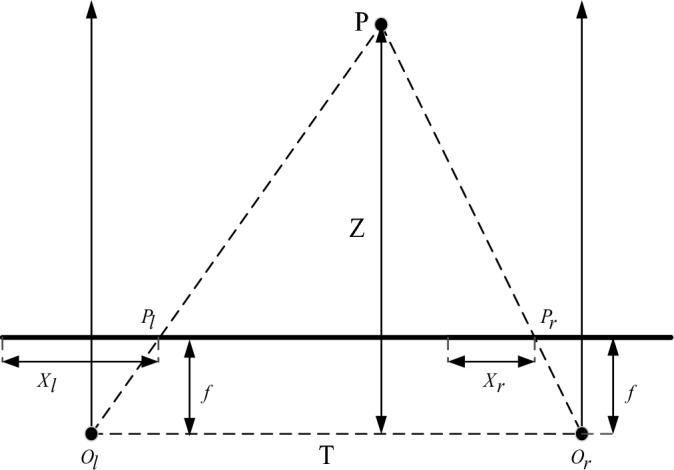
Binocular visual ranging principle.

Binocular distance measurement is a principle that simulates biological binocular distance measurement^8,9^. The left and right images are obtained by binocular camera, and the acquired images are transmitted to the computer for analysis and calculation of parallax, and then the three-dimensional spatial information of the target object is obtained. In an ideal situation, binocular distance measurement is obtained by two identical and parallel cameras, and the target distance information is calculated^10,11^. Its schematic diagram is shown in Figure 1: Suppose P is the target to be measured, O_l_ and O_r_ are the photocentroid of the left and right cameras, T is the photocentroid distance of the left and right cameras, also known as the baseline distance, f is the focal length of the camera, P_l_ and P_r_ are the coordinates of point P in the image coordinate system of the left and right cameras, and Z is the vertical distance from point P to the camera.

As can be seen from Fig. 1, $\Delta PP_{l} P_{r} \sim \Delta PO_{l} O_{r}$,formula (1) can be obtained:

1 $$ \frac{{T - (X_{l} - X_{r} )}}{T} = \frac{Z - f}{Z} $$

Then, the expression of distance Z between the target to be measured and the camera can be derived as follows: Formula (2):

2 $$ Z = \frac{fT}{{X_{l} - X_{r} }} = \frac{fT}{d} $$

where, $X_{l}$ and $X_{r}$ are the horizontal coordinates of pixel points in the left and right images of point P respectively, d is the parallax between the left and right cameras, $d = X_{l} - X_{r}$, and the focal length f and baseline distance T can be obtained by camera calibration. Therefore, the distance information of the target to be measured can be obtained only by obtaining parallax d.

**Figure 2:**
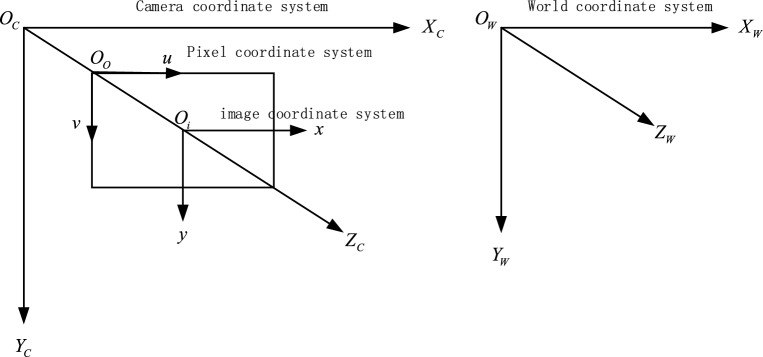
Relation diagram of the four coordinate systems.

In the imaging of binocular stereo vision system, it is mainly to convert spatial three-dimensional coordinates to pixel coordinates, and the relationship between the four coordinate systems involved is shown in Fig. 2. Where in, the image coordinate system $(x,y)$: the coordinate system takes the center of the image Oi as the origin, the x axis and y axis directions are consistent with the horizontal direction and vertical direction of the image respectively, and the physical length of the unit pixel in the x axis and y axis directions are $dx$ and $dy$ respectively. Pixel coordinate system $(u,v)$: The origin O_o_ of the coordinate system is the upper left corner of the image, and the u axis and v axis are parallel to the coordinate system of the image coordinate system, which is mainly used to describe the pixel position of a certain point in the image^12,13^. Camera coordinate system $(X_{C} ,Y_{C} ,Z_{C} )$: The coordinate system takes the optical center of the camera Oc as the origin, and the image coordinate system is a transmission projection relationship, so the X and Y axes of the coordinate system are consistent with the horizontal and vertical directions of the picture, and the Z axis is parallel to the optical axis of the lens^14,15^. The distance between the origin of the camera coordinate system and the origin of the plane coordinate system of the picture is the focal length of the camera. World coordinate system $(X_{W} ,Y_{W} ,Z_{W} )$: This coordinate system is an absolute coordinate system in real space to determine the relative position of the camera and the target in space, and the origin can be selected by oneself subjectively.

**Figure 3:**
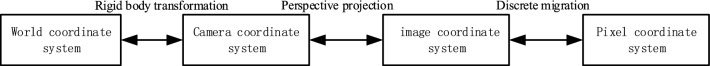
Transformation diagram of the four coordinate systems.

The conversion process between the four coordinate systems is shown in Figure 3:

If O_i_ is set as $(u_{0} ,v_{0} )$ in pixel coordinate system, the corresponding relation of a point on the imaging plane in pixel coordinate system $p(x,y)$ is shown in formula (3).

3 $$ \left\{ {\begin{array}{*{20}c} {u = \frac{x}{dx} + u_{0} } \\ {v = \frac{y}{dy} + v_{o} } \\ \end{array} } \right. $$

Written in matrix form as in formula (4):

4 $$ \left( {\begin{array}{*{20}c} u \\ v \\ 1 \\ \end{array} } \right) = \left( {\begin{array}{*{20}c} \frac{1}{dx} & 0 & {u_{0} } \\ 0 & \frac{1}{dy} & {v_{0} } \\ 0 & 0 & 1 \\ \end{array} } \right)\left( {\begin{array}{*{20}c} x \\ y \\ 1 \\ \end{array} } \right) $$

According to the triangular similarity principle, the corresponding relationship between point P $(X_{C} ,Y_{C} ,Z_{C} )$ on the space and point p on the imaging plane can be obtained as shown in formula (5):

5 $$ \left\{ \begin{gathered} x = f\frac{{X_{C} }}{{Z_{C} }} \hfill \\ y = f\frac{{Y_{C} }}{{Z_{C} }} \hfill \\ \end{gathered} \right. $$

where f is the focal length of the camera, then the homogeneous linear transformation form can be expressed as formula (6):

6 $$ Z_{C} \left( {\begin{array}{*{20}c} x \\ y \\ 1 \\ \end{array} } \right) = \left( {\begin{array}{*{20}c} f & 0 & {\begin{array}{*{20}c} 0 & 0 \\ \end{array} } \\ 0 & f & {\begin{array}{*{20}c} 0 & 0 \\ \end{array} } \\ 0 & 0 & {\begin{array}{*{20}c} 1 & 0 \\ \end{array} } \\ \end{array} } \right)\left( {\begin{array}{*{20}c} {X_{C} } \\ {Y_{C} } \\ {Z_{C} } \\ 1 \\ \end{array} } \right) $$

By substituting formula (6) into formula (4), the corresponding relationship between point P on space and pixel coordinate system can be obtained, as shown in formula (7):

7 $$ \left( {\begin{array}{*{20}c} u \\ v \\ 1 \\ \end{array} } \right) = \frac{1}{{Z_{C} }}\left( {\begin{array}{*{20}c} \frac{1}{dx} & 0 & {u_{0} } \\ 0 & \frac{1}{dy} & {v_{0} } \\ 0 & 0 & 1 \\ \end{array} } \right)\left( {\begin{array}{*{20}c} f & 0 & {\begin{array}{*{20}c} 0 & 0 \\ \end{array} } \\ 0 & f & {\begin{array}{*{20}c} 0 & 0 \\ \end{array} } \\ 0 & 0 & {\begin{array}{*{20}c} 1 & 0 \\ \end{array} } \\ \end{array} } \right)\left( {\begin{array}{*{20}c} {X_{C} } \\ {Y_{C} } \\ {Z_{C} } \\ 1 \\ \end{array} } \right) = \frac{1}{{Z_{C} }}\left( {\begin{array}{*{20}c} \frac{f}{dx} & 0 & {\begin{array}{*{20}c} {u_{0} } & 0 \\ \end{array} } \\ 0 & \frac{f}{dy} & {\begin{array}{*{20}c} {v_{0} } & 0 \\ \end{array} } \\ 0 & 0 & {\begin{array}{*{20}c} 1 & 0 \\ \end{array} } \\ \end{array} } \right)\left( {\begin{array}{*{20}c} {X_{C} } \\ {Y_{C} } \\ {Z_{C} } \\ 1 \\ \end{array} } \right) $$

The conversion between the world coordinate system and the camera coordinate system only needs to be realized through the rotation matrix R and the translation vector T^16,17^, then the corresponding relationship is shown in Formula (8):

8 $$ \left( {\begin{array}{*{20}c} {X_{C} } \\ {Y_{C} } \\ {Z_{C} } \\ 1 \\ \end{array} } \right) = \left( {\begin{array}{*{20}c} R & T \\ {0^{T} } & 1 \\ \end{array} } \right)\left( {\begin{array}{*{20}c} {X_{w} } \\ {Y_{w} } \\ {Z_{w} } \\ 1 \\ \end{array} } \right) $$

Then substitute formula (8) into formula (7) to obtain the correspondence between the world coordinate system and the pixel coordinate system, as shown in formula (9):

9 $$ \left( {\begin{array}{*{20}c} u \\ v \\ 1 \\ \end{array} } \right) = \frac{1}{{Z_{C} }}\left( {\begin{array}{*{20}c} \frac{f}{dx} & 0 & {\begin{array}{*{20}c} {u_{0} } & 0 \\ \end{array} } \\ 0 & \frac{f}{dy} & {\begin{array}{*{20}c} {v_{0} } & 0 \\ \end{array} } \\ 0 & 0 & {\begin{array}{*{20}c} 1 & 0 \\ \end{array} } \\ \end{array} } \right)\left( {\begin{array}{*{20}c} R & T \\ {0^{T} } & 1 \\ \end{array} } \right)\left( {\begin{array}{*{20}c} {X_{w} } \\ {Y_{w} } \\ {Z_{w} } \\ 1 \\ \end{array} } \right) $$

Simplified as formula (10):

10 $$ Z_{C} \left( {\begin{array}{*{20}c} u \\ v \\ 1 \\ \end{array} } \right) = M_{1} M_{2} \left( {\begin{array}{*{20}c} {X_{w} } \\ {Y_{w} } \\ {Z_{w} } \\ 1 \\ \end{array} } \right) $$

where, $M_{1}$ represents the camera internal parameter matrix; $M_{2}$ represents the camera external parameter matrix.

Underwater imaging model analysis

**Figure 4:**
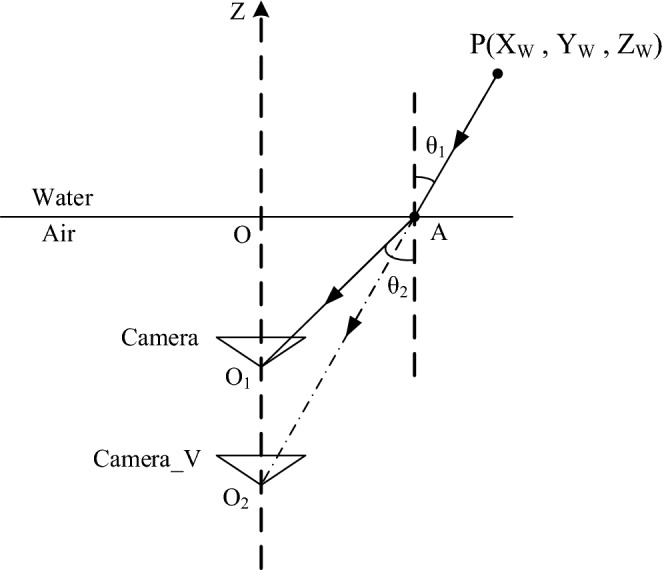
Underwater imaging model.

During the propagation of light from the underwater object to the camera lens, it undergoes two refractions, which occur at the interface between water and glass and the interface between glass and air. Because the glass is thin and uniform in texture, the refraction effect of the glass plate can be ignored^18,19^. Two refractions are equivalent to one refraction from water to air. For the convenience of the study, we assume that the optical axis of the camera is perpendicular to the refraction plane. After the above equivalence, the underwater imaging model can be simplified to Figure 4.

In Fig. 4, $P\left( {X_{W} ,Y_{W} ,Z_{W} } \right)$ is the target point to be measured, $O_{{1 }}$ is the real position of the camera, $O_{{2 }}$ is the intersection point of the extension line of incident light in water on the optical axis of the camera, which is taken as the virtual camera position, $O$ is the refraction point of incident light on the interface between water and air^20,21^, A is the intersection point of the camera optical axis and the interface between water and air, $\theta_{{1 }}$ and $\theta_{{2 }}$ are respectively the incidence Angle and the refraction Angle.

$n_{{1}}$ and $n_{{2 }}$ are the refractive indices of water and air respectively. According to triangle similarity and refraction law $n_{{1 }} \sin \theta_{{1 }} = n_{{2 }} \sin \theta_{{2 }}$, it can be obtained:

11 $$ \frac{{\sin \theta_{{1 }} }}{{\sin \theta_{{2 }} }} = \frac{{n_{{2 }} }}{{n_{{1}} }} $$

Because the optical axis of the real camera and the virtual camera coincide, the projection length of $OA$ on the two cameras is the same, that is, $X_{{1 }} = X_{{2 }}$, $X_{{1 }}$ and $X_{{2 }}$ are the imaging lengths of $OA$ in the real camera and the virtual camera, respectively. As a result:

12 $$ \frac{{X_{{1 }} }}{OA} = \,\frac{{f_{1} }}{{OO_{1} }} $$

13 $$ \frac{{X_{{2 }} }}{OA} = \,\frac{{f_{2} }}{{OO_{2} }} $$

According to formulas (12) and (13):

14 $$ \frac{{f_{1} }}{{f_{2} }} = \,\frac{{OO_{1} }}{{OO_{2} }}\,\frac{{\tan \theta_{1} }}{{\tan \theta_{{_{2} }} }} $$

Equations (11) and (14), we get:

15 $$ \frac{{f_{1} }}{{f_{2} }} = \sqrt {\frac{{\left( {\left( {n_{2} /n_{1} } \right)^{2} - \sin^{2} \theta_{1} } \right)}}{{1 - \sin^{2} \theta_{1} }}} $$

When the Angle between the light and the normal is small, the virtual focal length of the camera can be calculated by formula (16):

According to the above analysis, it is found that the actual focal length of the camera under water changes, and in a certain range, this change can be regarded as a linear change. Therefore, when the camera is used underwater, the focal length of the virtual camera can be obtained according to the refractive index. In other words, under normal circumstances, the focal length obtained by the water calibration should be 1.33 times that of the air^22^.

**Figure 5:**
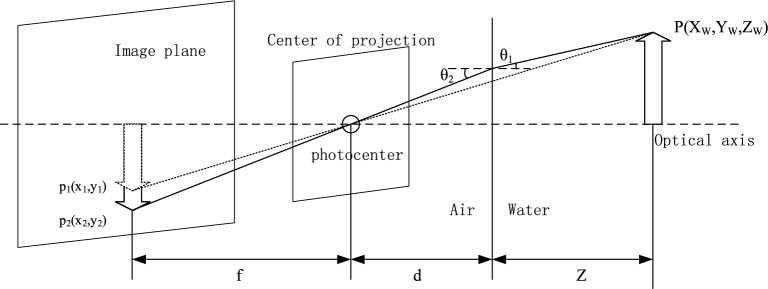
Diagram of distortion caused by underwater refraction.

The camera itself is biased during the manufacturing and installation process, and the effect of underwater refraction on image imaging can produce even greater distortion. Figure5 shows the schematic diagram of imaging deviation caused by underwater refraction^23^.

In the figure, $P$ is the three-dimensional coordinate point, $p_{2}$ is the actual imaging point, $p_{1}$ is the ignored refraction imaging point, $f$ is the focal length, $d$ is the distance from the camera to the waterproof plane,$\theta_{1}$ and $\theta_{2}$ are respectively the incidence Angle and the refraction Angle. The following relationship can be obtained through geometric relations:

17 $$ x_{1} = f\frac{{X_{W} }}{Z + d} $$

18 $$ x_{2} = f\tan \theta_{2} $$

From formulas (17) and (18):

19 $$ \frac{{x_{2} }}{{x_{1} }} = \tan \theta_{2} \frac{{\left( {Z + d} \right)}}{{X_{W} }} $$

Since $X_{W} \gg d$, according to $n_{{1}} \sin \theta_{{1}} = n_{{2}} \sin \theta_{{2}}$, we can get:

20 $$ \frac{{x_{r} }}{{x_{v} }} = \frac{{\tan \theta_{2} }}{{\tan \theta_{1} }} = \frac{{\tan \theta_{2} }}{{\tan \left( {\arcsin \left( {\frac{{n_{2} \sin \theta_{2} }}{{n_{1} }}} \right)} \right)}} $$

It can be found that the distance between the imaging point and the imaging center and the distance between the luminous point and the optical axis is no longer a linear relationship in the pinhole imaging model, but a nonlinear relationship, which causes imaging deviation and intensifies image distortion, and the distortion becomes more obvious with the increase of distance.

Through the above analysis, it is found that the deviation caused by underwater refraction can be partially corrected by equivalent focal length change. However, this deviation is not linear, and for the nonlinear part, it needs to be corrected by using the image distortion correction polynomial.

According to underwater imaging analysis, it is found that underwater refraction has two main effects on the camera:

- (1) The equivalent focal length of the camera changes, and the change in focal length can be regarded as the product of the original focal length and the ratio of refractive index in a certain Angle of view;
- (2) The image distortion is intensified, so that the previous distortion correction does not meet the underwater use, and the distortion increases with the increase of the distance from the imaging center, similar to the pillow distortion.

In order to ensure the accuracy of underwater target ranging and size measurement, the following formula is used to correct the distortion and ensure the measurement accuracy of the system.

(1) As the distance from the imaging point to the image center increases, the radial distortion increases accordingly, so the quadratic and higher-order polynomial functions related to the distance are used to correct the radial distortion. The radial distortion correction formula is as follows:

21 $$ \left\{ {\begin{array}{*{20}c} {x_{r} = x(1 + k_{1} r^{2} + k_{2} r^{4} + k_{3} r^{6} )} \\ {y_{r} = y(1 + k_{1} r^{2} + k_{2} r^{4} + k_{3} r^{6} )} \\ \end{array} } \right. $$

In the formula, $(x,y)$ is the coordinate of the distortion point in the image before correction, $(x_{r} ,y_{r} )$ is the coordinate of the distortion point in the image after correction, r is the distance from the distortion point to the image center, and $k_{1}$, $k_{2}$ and $k_{3}$ are the radial distortion coefficients.

(2) The optical axis of the lens is not perpendicular to the image plane, causing tangential distortion. Tangential distortion correction formula is as follows:

22 $$ \left\{ {\begin{array}{*{20}c} {x_{t} = x + 2p_{1} xy + p_{2} (r^{2} + 2x^{2} )} \\ {y_{t} = y + p_{1} (r^{2} + 2y^{2} ) + 2p_{2} xy} \\ \end{array} } \right. $$

In the formula, $p_{1}$ and $p_{2}$ are tangential distortion coefficients.

Image semantic segmentation based on Deeplabv3+

Semantic segmentation is an important technique in the field of computer vision, which aims to assign each pixel in an image to a specific semantic category^24,25^. Unlike ordinary image segmentation, semantic segmentation does not just divide the image into different areas, but requires the classification of each pixel so that each pixel can be assigned a specific semantic label, such as people, cars, roads, trees, etc. This fine pixel-level classification can provide a richer and more detailed understanding of images, providing an important foundation for many computer vision tasks^26,27^.

DeepLabV3+, UNet, FCN, and PSPNet are all commonly used semantic segmentation models with some differences in network structure and performance. Using encoder-decoder structure, UNet network can effectively extract image features and restore image resolution, but the model parameters are too large, the training and reasoning speed is slow, and the semantic segmentation effect is not ideal for some complex scenes. FCN network uses the full convolutional network structure to classify the entire image at the pixel level. Because it does not consider the fusion of multi-scale features and the use of context information, the semantic segmentation effect is not ideal for some complex scenes, and the detection effect is not ideal for some small targets. PSPNet network adopts pyramid pool module, which can capture features of different scales and improve the model's receptor field and context information. Because the fusion of multi-scale features is not considered, the semantic segmentation effect in some complex scenes is not ideal, and the detection effect in some small targets is not ideal. DeepLabV3+ uses hollow convolution and multi-scale feature fusion, which can effectively improve the accuracy and robustness of semantic segmentation. ASPP module can capture features of different scales and improve the receptive field and context information of the model. At the same time, DeepLabV3+ can be equipped with lightweight backbone network MobileNetv2, which has high detection accuracy and speed. The experimental results show that the detection accuracy can reach more than 88% and the detection speed can reach 89FPS, which fully meets the real-time requirements of ROV underwater detection.

**Figure 6:**
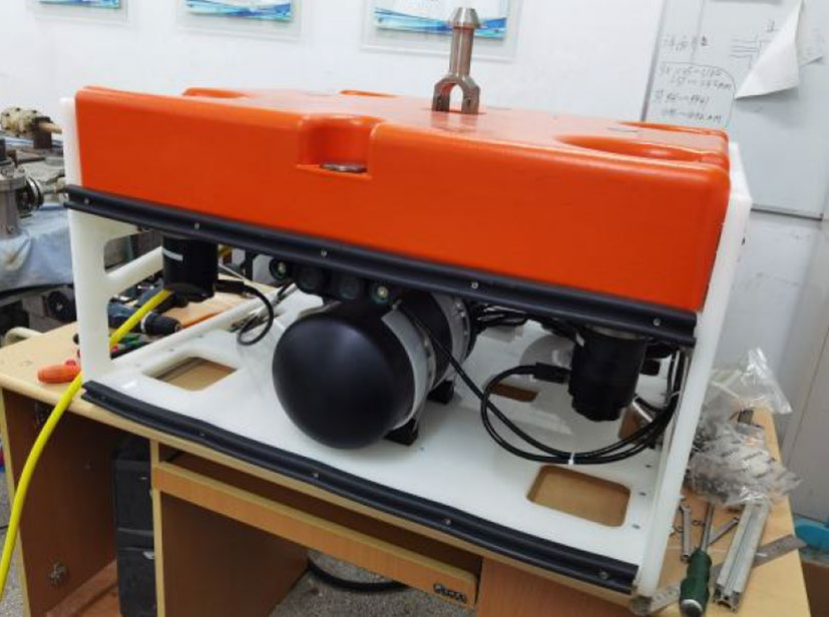
Underwater robot.

The DeepLabv3+ model reduces the size and calculation amount of the model while maintaining high accuracy, so that it is more suitable for deployment and application on underwater vehicles. Figure 6 shows the binocular camera of the underwater robot photographing the steel pipe target in the laboratory environment.

**Figure 7:**
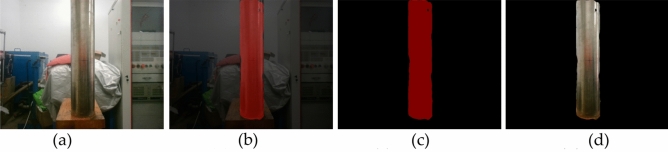
Semantic segmentation of visual images, (**a**) Original drawing, (**b**) Hybrid graph, (**c**), Spanning graph, (**d**) Object map.

The image after semantic segmentation can generally be visualized in three ways, as shown in b, c and d in Figure 7. The steel pipe in Figure 7a is the underwater detection target simulated by us; Figure 7b is a mixed image of segmentation target and image background. The background of the mixed image is dark, and all segmentation targets are marked in red^28,29^. In Figure 7c, the background is deleted and only the segmented target is retained. All the steel pipe parts are marked in red. Figure 7d is the object map with the background removed and only the segmented object retained. Complex images may require more computing resources, resulting in slower operations. Figure 7c and Figure 7d are deducted from the background, the use of images is more concise, the contrast is better, saving computing resources, improve the stereo matching speed.

In binocular vision, stereo matching is the process of finding the corresponding points in the images of the left and right cameras. The traditional stereo matching algorithm will search the whole image range, which is computationally heavy and easy to be disturbed by background noise. However, after using semantic segmentation, pixel matching only needs to be limited to the segmentation Region, that is, focusing on region ROI (Region of Interest), and the parallax search range is only within ROI when calculating the global energy function. The algorithm only calculates and matches parallax in this region, thus reducing the computation and improving the processing efficiency. That Narrows the search area considerably. As can be seen from Figure 7d, the target map of the semantic segmentation image only contains the outline of the pile leg, and the background information is removed. Only the pixels on the pile leg need to be considered during matching, thus reducing the matching search range and improving the matching speed. Although Figure 7c only shows the pile leg region, all of them are marked in red, resulting in the same features of all pixels in the pile leg, without any difference, and the pixel blocks cannot be correctly matched, resulting in large measurement errors, so they cannot be used.

It is assumed that the semantic segmentation images captured by the left camera and the right camera are L and R respectively, the corresponding pixels are $(x_{l} ,y)$ and $(x_{r} ,y)$, and the parallax is d. The width of the entire image is W, the number of pixels on the pile leg is N, the time to match each pixel is $t_{m}$, and the time to match the entire pile leg can be expressed as:

23 $$ T_{{{\text{p}}ile - leg}} = N \times t_{m} $$

In the case of the original image, all pixels of the entire image need to be matched, and the matching time can be expressed as:

24 $$ T_{total} = W \times t_{m} $$

The proportion of semantic segmentation image matching time to original image matching time is:

25 $$ P\% = \frac{{T_{pile - leg} }}{{T_{total} }} = \frac{{N \times t_{m} }}{{W \times t_{m} }} = \frac{N}{W} \times 100\% $$

By removing the background and keeping only the target object, the matching time will be greatly reduced. Taking Figure 7 as an example, the matching time of the original image is only about 1/7.

### Binocular ranging experiment

**Figure 8:**
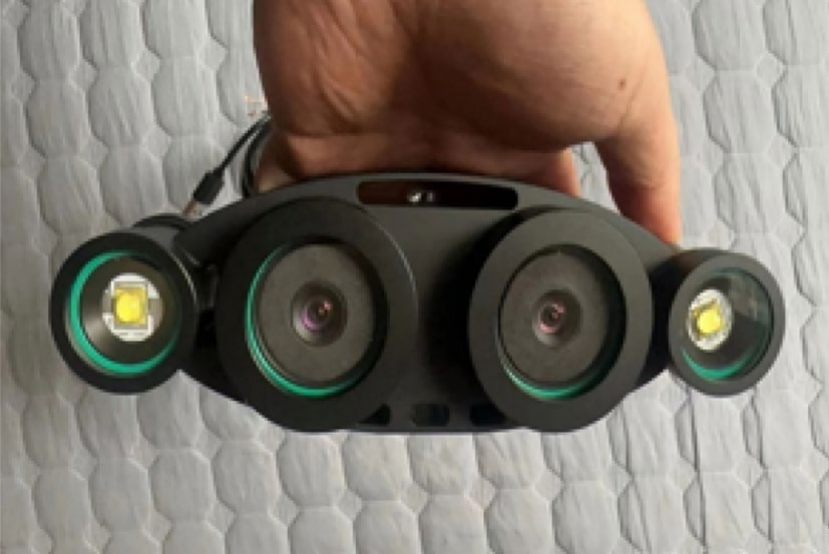
Underwater HD binocular camera.

Binocular ranging experiment environment and hardware used in the experiment: desktop computer and underwater high-definition binocular camera are shown in Figure 8. The desktop computer configuration is as follows: CPU is Intel i7, memory is 32GB, GPU is NVIDIA GeForce RTX 3070; Underwater binocular camera configuration: underwater high-definition binocular camera, image resolution 1920×960, baseline length 60mm. The software environment is windows 10, Matlab 2021b, OpenCV.

**Figure 9:**
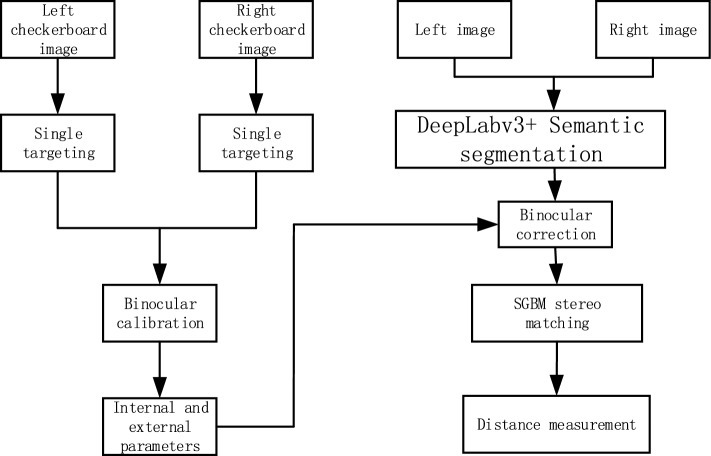
Flow chart of target ranging based on binocular vision.

The target ranging process based on binocular vision is shown in Figure 9. Firstly, matlab toolbox is used to calibrate the left and right cameras, and the left and right checkerboard images are imported to carry out single target calibration and binocular calibration respectively, and the internal and external parameters of binocular cameras are obtained. After that, the Deeplabv3+ model is semantically segmpled to the images captured by the binocular camera, and the generated map with the background removed is obtained^30^. Finally, the two eyes of the image generated by semantic segmentation are corrected, and three-dimensional matching is carried out by SGBM method. A point is selected on the simulated pile leg, and the distance between the ROV and the selected point of the target object is measured.

### Calibration and alignment of binocular cameras

**Figure 10:**
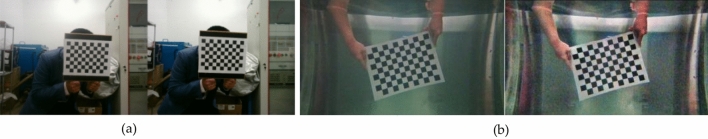
Image calibration, (**a**) Airborne calibration (**b**) Underwater calibration.

In this paper, Zhang Zhengyou calibration method is used to calibrate binocular cameras^31^. First, an 8 ×11 checkerboard calibration board image was made, the size of each square was 22mm×22mm, and it was fixed on the cardboard. Open the binocular camera, and capture 23 sets of checkerboard images at different angles in the air and water tank by constantly moving the calibration plate, Then the camera is calibrated by calculating and analyzing the corners of the checkerboard (solving the internal and external parameters of the camera).as shown in Fig. 10.

**Figure 11:**
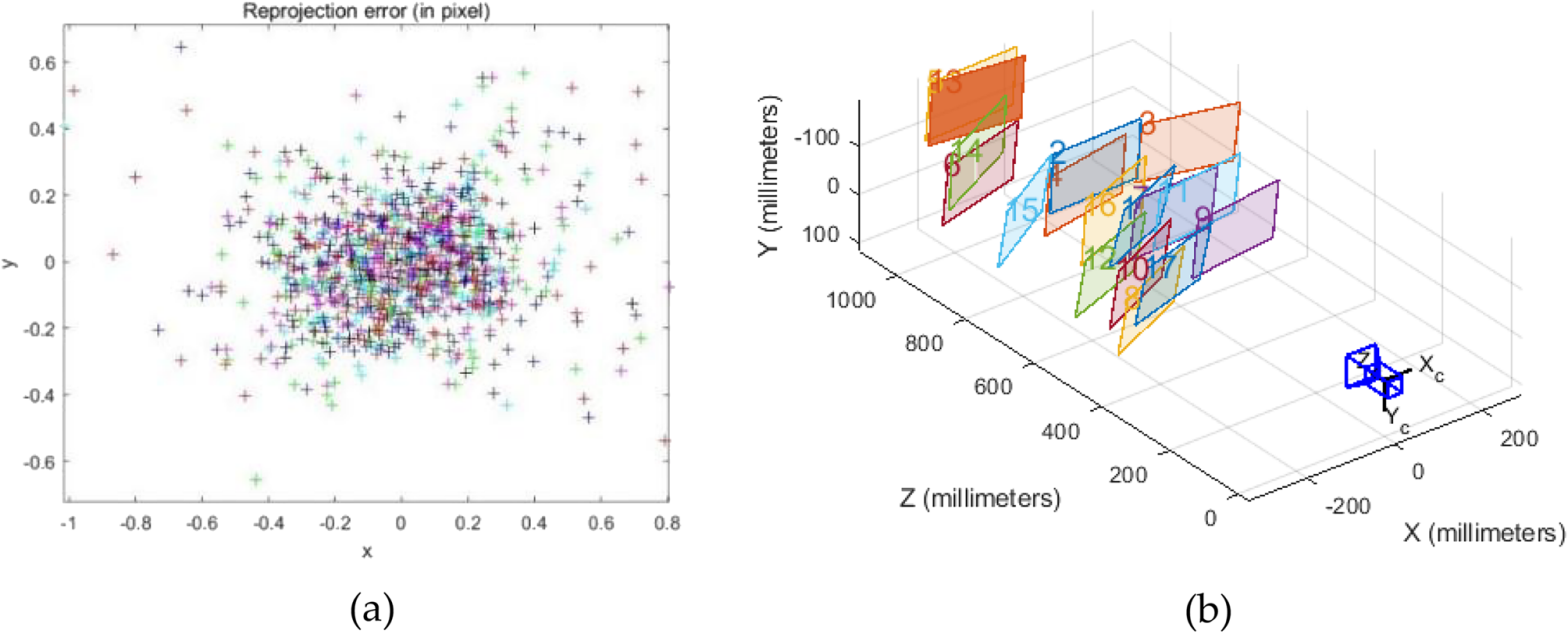
Single objective rendering, (**a**) Corner error distribution diagram (**b**) Single camera with calibration board position 3D view.

In Matlab, 20 groups of calibration images are read in turn, the corner of each calibration image is accurately extracted clockwise from the upper left corner of the calibration image, and the parameters of the monomer camera are obtained, and the corner error distribution Fig. 11a and the 3D view of the position of a single camera and the calibration board Fig. 11b are obtained. It can be seen that most corner points are distributed between [-0.4,0.4], with dense distribution and high precision^32^.

The calibration parameters of the left and right monocular cameras were input into Matlab, and the binocular cameras were continued to be calibrated. The calibration parameters of the binocular camera in air and binocular camera in water were obtained respectively, as shown in Table 1 and Table 2. The air and underwater camera parameter data in Table 1 and Table 2 were compared and analyzed. The actual baseline of the camera was 60mm, the underwater calibration baseline was 58.6325mm, and the air calibration baseline was 59.2756mm, all of which were within the acceptable range. The fx and fy of the left camera are 852.875 and 851.195 in water, which are quite different from the calibration results in air. According to the above analysis, the focal length of the camera in water is about 1.33 times that in air under certain circumstances, and the experimental data,852.8751/634.1084=1.344 and 851.193/631.9197 =1.346, are all about 1.33, thus verifying the correctness of the above analysis. By comparing the distortion coefficient of the camera, it can be seen that the distortion coefficient of the underwater camera is greater than that of the air, which is also in line with the above analysis of the influence of underwater refraction on camera distortion.

**Table 1:** Calibration parameters of binocular camera in air.

Argument		Left camera			Right camera	
Internal parameter matrix	634.1084	1.1661	401.3114	635.3628	1.0953	388.3334
	0	631.9197	305.6509	0	633.1738	299.6141
	0	0	1	0	0	1
Radial distortion	− 0.0724	0.4413	− 0.6813	− 0.0684	0.3424	− 0.4191
Tangential distortion	0		0	0		0
Rotation matrix	1		0.0015		− 0.0036	
	− 0.0014		0.9999		0.0110	
	0.0036		− 0.0110		0.9999	
Translation vector	− 59.2796		− 0.2593		1.1542

**Table 2:** Calibration parameters of underwater binocular camera.

Argument		Left camera			Right camera	
Internal parameter matrix	852.8751	1.1661	401.5783	864.0935	1.0953	388.9654
	0	851.1953	304.2632	0	867.1186	299.8731
	0	0	1	0	0	1
Radial distortion	− 0.1325	0.6658	− 0.3654	− 0.1368	0.6721	− 0.3588
Tangential distortion	0		0	0		0
Rotation matrix	0.9999		2.6179			− 0.0069
	− 2.8941		0.9999			3.2314
	0.0058		− 0.0110			0.9999
Translation vector	− 58.6325		− 1.1842			1.6924

**Figure 12:**
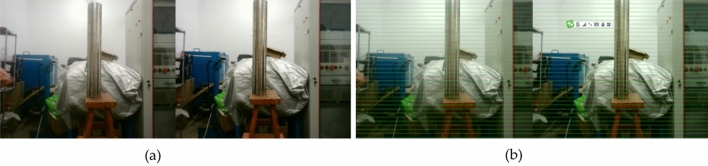
Before and after binocular correction. (**a**) Binocular vision image before correction (**b**) binocular vision image after correction.

After the camera calibration is completed, the OpenCV computer vision library is used to perform binocular alignment. The comparison between the effect before and after correction is shown in Figure 12.

### Binocular ranging

**Figure 13:**
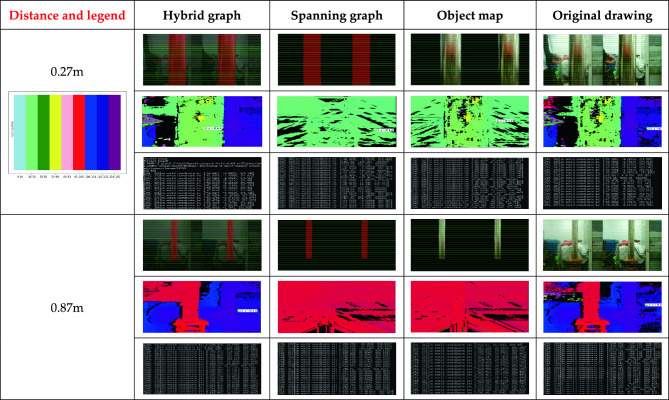
Comparison of binocular ranging effect of different semantic segmentation graphs.

Under the condition that the camera position is fixed, a steel pipe is placed in front of the binational camera of the underwater robot, and images are taken at different distances in order in both air and underwater environments. The Deeplabv3+ model is used to conduct semantic segmentation of the above images, and the hybrid image of target and background recognition and the generated image of target recognition are generated respectively. Only the object map that identifies the object and removes the background. SGBM algorithm is used to carry out distance measurement experiments on the same pixel coordinates of the above three semantic segmentation maps and the original images, and finally the distance information of the target is obtained by using the binocular distance measurement formula. The actual effect of target distance measurement based on binocular vision and semantic segmentation is shown in Figure 13, showing the distance measurement effect of 0.27m and 0.87m.The image in the first row of the same measured distance in Figure 13 is the stereo matching original. The image in the second row is the parallax map generated after stereo matching. In the left and right binocular images, parallax refers to the horizontal distance between the center pixels of the two matching blocks, which reflects the depth information of the object in three-dimensional space. Each pixel value represents the parallax size of the corresponding point. The magnitude of the parallax is inversely proportional to the distance of the object from the camera. That is, the larger the parallax value, the closer the object is to the camera. In a parallax chart, different colors represent different parallax values. The image in the third row is the calculated distance value for each pixel.

The measurement results of different distances and images in the air are shown in Table 3, and the measurement results of different distances and images under water are shown in Table 4.

**Table 3:** binocular ranging data in the air.

Actual distance (m)	Mixed graph measurements (m)	Relative error	Generate graph measurements (m)	Relative error	Target map measurements (m)	Relative error	Measurements of the original drawing (m)	Relative error
0.27	0.2715	0.55%	0.3204	18.66%	0.2708	0.29%	0.2708	0.29%
0.87	0.8872	1.97%	0.9255	6.37%	0.8834	1.54%	0.8834	1.54%
0.97	1.0075	3.86%	1.0127	4.40%	1.0062	3.73%	1.0062	3.73%
1.12	1.1796	5.32%	1.1863	5.91%	1.177	5.08%	1.177	5.08%
Measuring time	0.1s		0.07s		0.07s		0.1s

**Table 4:** binocular ranging data in water.

Actual distance (m)	Mixed graph measurements(m)	Relative error	Generate graph measurements(m)	Relative error	Target map measurements(m)	Relative error	Measurements of the original drawing(m)	Relative error
0.30	0.3048	1.60%	0.3165	5.50%	0.3036	1.20%	0.3036	1.20%
0.55	0.5378	2.21%	0.5025	9.54%	0.5604	1.89%	0.5604	1.89%
0.80	0.7714	3.57%	0.7532	5.85%	0.7863	2.96%	0.7863	2.96%
0.90	0.8568	4.80%	0.8371	6.98%	0.8625	4.68%	0.8625	4.68%
Measuring time	0.1s		0.07s		0.07s		0.1s	

As can be seen from the binocular ranging data in Table 3 and Table 4 above, the distance information calculated by SGBM stereoscopic matching has relatively small error with the real distance during short-range ranging, and has high accuracy. In the long distance distance, the error is relatively large, the accuracy becomes low. The reason for this phenomenon is that when the target is relatively close to the camera, the target has more effective pixels in the imaging plane, and these pixels will be simpler and more accurate to find the best matching point, so the accuracy of the ranging is higher; However, when the target is farther and farther from the camera, the effective pixels of the target in the imaging plane become less, the matching difficulty increases, and the matching error becomes larger, so the accuracy of the ranging is correspondingly lower. The greater the actual distance, the greater the relative error.

Since the pixels of target recognition are all changed to red in the mixed map and the generated map, the target area is exactly the same without difference, and the target recognition area is all the same and can be matched, resulting in calculation errors, which should not be used in actual distance measurement. The ranging results of the target map and the original map are exactly the same. The mixed map and the generated map change the recognition area of the target uniformly to red, resulting in the disappearance of the image features of the underwater pile leg, which can not be accurately matched in stereo, and thus cause a large error in the ranging results. The stereo matching effect between the target image and the original image is the best, and the error is small. The mixed image and the original image have high complexity, long stereo matching time, and the ranging time is 0.1s/ time. The generated image and the target image reduce the complexity of the image and improve the stereo matching speed because the background is removed. The ranging time is 0.07s/ time, and the ranging speed is increased by 30%. Therefore, using the generated target map to measure the distance after the underwater object is identified not only improves the measurement speed, but also ensures that the measurement accuracy is not reduced.

### The influence of underwater environment on binocular rangingg

**Figure 14:**
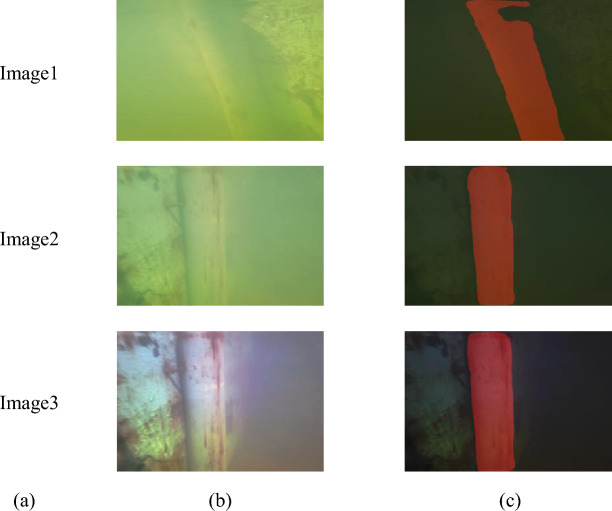
Influence of underwater environment on target recognition results. (**a**) Picture name (**b**) Underwater target map (**c**) Underwater target identification map.

In the real underwater environment, binocular vision ranging will be affected by many factors such as illumination and environmental scattering. Such as suspended particles and water molecules. These scattering media will cause the light to scatter during the propagation process, making the image blurred and distorted. As shown in Figure 14, the scattering phenomenon of Image1 and Image2 is serious, resulting in blurred images, and the recognition accuracy of Deeplabv3+ is also decreased. There is a large gap in the recognition area of Image1, and the segmentation of the right edge line of the target object in Image2 is not smooth, thus increasing the error and uncertainty of ranging. The binocular vision system may not be able to accurately match the corresponding pixels, thus affecting the accuracy of the ranging results. Image3 is an enhanced image of Image2, and its right edge line segmentation effect is better than Image2, and the right edge line is smoother.

Table 5 shows the influence of different underwater environments on binocular ranging results. Clear underwater images are superior to fuzzy underwater images in both accuracy and speed. The weakening of underwater light intensity and scattering phenomenon make the edges and details of objects blurred, and the number of pixels occupied by the target in the image will be reduced, resulting in a decrease in pixel density. Lower pixel densities can result in more difficulty in accurately extracting and matching target features, reducing the accuracy of ranging. If the image is blurred, the system needs to spend more time to search and match the corresponding feature points, which will increase the time of stereo matching and reduce the measurement speed. In addition, the instability of underwater illumination direction may also lead to misjudgment of binocular vision system, which further affects the accuracy of ranging.

**Table 5:** Comparison table of underwater image ranging data with different resolution.

Measurement image	True distance (m)	Measuring distance (m)	Relative error (%)	Measurement time (s)
Image2	1.0	0.9542	4.58	0.073
Image3	1.0	0.9664	3.36	0.071

The advantages of using segmentation image to calculate parallax compared with using original image are mainly reflected in the following aspects:

- (1) Improve the accuracy of the calculation: the segmentation image can limit the scope of the calculation parallax to the target object, avoiding background interference, thus improving the accuracy of the calculation.
- (2) Improve computing efficiency: Segmentation of images can reduce the number of pixels to calculate parallax, thus improving the efficiency of calculation.
- (3) Enhance the understanding of the target object: segmented images can provide the semantic information of the target object, which is conducive to the understanding and analysis of the target object.
- In general, the calculation of parallax using segmentation image can improve the accuracy and efficiency of the calculation and enhance the understanding of the target object compared with the calculation of parallax using ordinary image.

## Conclusions

Aiming at the technical requirements of target detection and target ranging in underwater vehicle operation, this paper designs a set of underwater vehicle target ranging system based on Deeplabv3+ semantic segmentation and binocular vision. The system uses Zhang Zhengyou calibration method to calibrate the binocular camera, and uses SGBM algorithm to carry out stereoscopic matching of binocular images and measure the distance. Through ranging experiments in air and water, the target object is less than 1m, and the measurement error is less than 5%. However, due to the scattering and refraction of light in the underwater environment, interference factors in the imaging plane will increase with the increase of distance. The difficulty of binocular stereo matching is increased, the matching accuracy is reduced, and the measurement error is increased correspondingly. At the same time, it is also verified that the accuracy of the measurement is unchanged and the measurement speed is increased by nearly 30% by using the target map after semantic segmentation.

## Data Availability

All data generated or analysed during this study are included in this published article.
