# Author Correction: Research on underwater robot ranging technology based on semantic segmentation and binocular vision

**DOI:** 10.1038/s41598-024-64858-z

**Published:** 2024-06-21

**Authors:** Qing Hu, Kekuan Wang, Fushen Ren, Zhongyang Wang

**Affiliations:** 1 Sanya Offshore Oil and Gas Research Institute, Northeast Petroleum University, Sanya, 572025 China; 2 CNPC Engineering Technology Research Company Limited, Tianjin, 300451 China

Correction to: *Scientific Reports* 10.1038/s41598-024-63017-8, published online 29 May 2024

In the original version of this Article, Qing Hu was incorrectly listed as a corresponding author. The correct corresponding author for this Article is Zhongyang Wang. Correspondence and request for materials should be addressed to wzy1719393666@126.com.

The original Article has been corrected.

